# An Effective Hybrid Firefly Algorithm with Harmony Search for Global Numerical Optimization

**DOI:** 10.1155/2013/125625

**Published:** 2013-11-20

**Authors:** Lihong Guo, Gai-Ge Wang, Heqi Wang, Dinan Wang

**Affiliations:** ^1^Changchun Institute of Optics, Fine Mechanics and Physics, Chinese Academy of Sciences, Changchun 130033, China; ^2^School of Computer Science and Technology, Jiangsu Normal University, Xuzhou, Jiangsu 221116, China

## Abstract

A hybrid metaheuristic approach by hybridizing harmony search (HS) and firefly algorithm (FA), namely, HS/FA, is proposed to solve function optimization. In HS/FA, the exploration of HS and the exploitation of FA are fully exerted, so HS/FA has a faster convergence speed than HS and FA. Also, top fireflies scheme is introduced to reduce running time, and HS is utilized to mutate between fireflies when updating fireflies. The HS/FA method is verified by various benchmarks. From the experiments, the implementation of HS/FA is better than the standard FA and other eight optimization methods.

## 1. Introduction

In engineering problems, optimization is to look for a vector that can maximize and minimize a function. Nowadays, stochastic method is generally utilized to cope with optimization problems [1]. Though there are many ways to classify them, a simple one is used to divide them into two groups according to their nature: deterministic and stochastic. Deterministic algorithms can get the same solutions if the initial conditions are unchanged, because they always follow the rigorous move. However, regardless of the initial values, stochastic ones are based on certain stochastic distribution; therefore they generally generate various solutions. In fact, both of them can find satisfactory solutions after some generations. Recently, nature-inspired algorithms are well capable of solving numerical optimization problems more efficiently. 

These metaheuristic approaches are developed to solve complicated problems, like permutation flow shop scheduling [2], reliability [3, 4], high-dimensional function optimization [5], and other engineering problems [6, 7]. In the 1950s, nature evolution was idealized as an optimization technology and this made a new type of approach, namely, genetic algorithms (GAs) [8]. After that, many other metaheuristic methods have appeared, like evolutionary strategy (ES) [9, 10], ant colony optimization (ACO) [11], probability-based incremental learning (PBIL) [12], big bang-big crunch algorithm [13–16], harmony search (HS) [17–19], charged system search (CSS) [20], artificial physics optimization [21], bat algorithm (BA) [22, 23], animal migration optimization (AMO) [24], krill herd (KH) [25–27], differential evolution (DE) [28–31], particle swarm optimization (PSO) [32–35], stud GA (SGA) [36], cuckoo search (CS) [37, 38], artificial plant optimization algorithm (APOA) [39], biogeography-based optimization (BBO) [40], and FA method [41, 42]. 

As a global optimization method, FA [42] is firstly proposed by Yang in 2008, and it is originated from the fireflies swarm. Recent researches demonstrate that the FA is quite powerful and relatively efficient [43]. Furthermore, the performance of FA can be improved with feasible promising results [44]. In addition, nonconvex problems can be solved by FA [45]. A summarization of swarm intelligence containing FA is given by Parpinelli and Lopes [46].

On the other hand, HS [17, 47] is a novel heuristic technique for optimization problems. In engineering optimization, the engineers make an effort to find an optimum that can be decided by an objective function. While, in the music improvisation process, musicians search for most satisfactory harmony as decided by aesthetician. HS method originates in the similarity between them [1]. 

In most cases, FA can find the optimal solution with its exploitation. However, the search used in FA is based on randomness, so it cannot always get the global best values. On the one hand, in order to improve diversity of fireflies, an improvement of adding HS is made to the FA, which can be treated as a mutation operator. By combining the principle of HS and FA, an enhanced FA is proposed to look for the best objective function value. On the other hand, FA needs much more time to search for the best solution and its performance significantly deteriorates with the increases in population size. In HS/FA, top fireflies scheme is introduced to reduce running time. This scheme is carried out by reduction of outer loop in FA. Through top fireflies scheme, the time complexity of HS/FA decreases from O(NP^2^) to O(KEEP∗NP), where KEEP is the number of top fireflies. The proposed approach is evaluated on various benchmarks. The results demonstrate that the HS/FA performs more effectively and accurately than FA and other intelligent algorithms.

The rest of this paper is structured below. To begin with, a brief background on the HS and FA is provided in Sections 2 and 3, respectively. Our proposed HS/FA is presented in Section 4. HS/FA is verified through various functions in Section 5, and Section 6 presents the general conclusions.

## 2. HS Method

As a relative optimization technique, there are four optimization operators in HS [17, 48, 49]:

 HM: the harmony memory, as shown in (1);
 HMS: the harmony memory size,
 HMCR: the harmony memory consideration rate,
 PAR*:* the pitch adjustment rate, and
 bw: the pitch adjustment bandwidth [1].

Consider

(1) $$\begin{array}{c} \text{HM}=\left[\begin{array}{cccc} x_{1}^{1} & x_{2}^{1} & \cdots & x_{D}^{1}\\ x_{1}^{2} & x_{2}^{2} & \cdots & x_{D}^{2}\\ \vdots & \vdots & \cdots & \vdots \\ x_{1}^{\text{HMS}} & x_{2}^{\text{HMS}} & \cdots & x_{D}^{\text{HMS}} \end{array}|\begin{array}{c} \text{fitness}\;\left(x^{1}\right)\\ \text{fitness}\;\left(x^{2}\right)\\ \vdots \\ \text{fitness}\;\left(x^{\text{HMS}}\right) \end{array}\right]. \end{array}$$

The HS method can be explained according to the discussion of the player improvisation process. There are 3 feasible options for a player in the music improvisation process: (1) play several pitches that are the same with the HMCR; (2) play some pitches like a known piece; or (3) improvise new pitches [1]. These three options can be idealized into three components: use of HM, pitch adjusting, and randomization [1]. 

Similar to selecting the optimal ones in GA, the first part is important as it is [1]. This can guarantees that the optimal harmonies will not be destroyed in the HM. To make HS more powerful, the parameter HMCR should be properly set [1]. Through several experiments, in most cases, HMCR = 0.7*∼*0.95.

The pitch in the second part needs to be adjusted slightly; and hence a proper method is used to adjust the frequency [1]. If the new pitch *x*_new_ is updated by

(2) $$\begin{array}{c} x_{\text{new}}=x_{\text{old}}+bw\left(2\varepsilon -1\right), \end{array}$$

where *ε* is a random number in [0,1] and *x*_old_ is the current pitch. Here, *bw* is the bandwidth.

Parameter PAR should also be appropriately set. If PAR is very close to 1, then the solution is always updating and HS is hard to converge. If it is next to 0, then little change is made and HS may be premature. So, here we set PAR = 0.1*∼*0.5 [1]. 

To improve the diversity, the randomization is necessary as shown in the third component. The usage of randomization allows the method to go a step further into promising area so as to find the optimal solution [1].

The HS can be presented in Algorithm 1. Where *D* is the number of decision variables. rand is a random real number in interval (0,1) drawn from uniform distribution.

## 3. FA Method

FA [42] is a metaheuristic approach for optimization problems. The search strategy in FA comes from the fireflies swarm behavior [50]. There are two significant issues in FA that are the formulation of attractiveness and variation of light intensity [42].

For simplicity, several characteristics of fireflies are idealized into three rules described in [51]. Based on these three rules, the FA can be described in Algorithm 2.

For two fireflies *x*_*i*_ and *x*_*j*_, they can be updated as follows:

(3) $$\begin{array}{c} x_{i}^{t+1}=x_{i}^{t}+\beta_{0}e^{-\gamma r_{ij}^{2}}\left(x_{i}^{t}-x_{j}^{t}\right)+\alpha \varepsilon_{i}^{t}, \end{array}$$

where *α* is the step size, *β*_0_ is the attractiveness at *r* = 0, the second part is the attraction, while the third is randomization [50]. In our present work, we take *β*_0_ = 1, *α* ∈ [0, 1], and *γ* = 1 [50].

## 4. HS/FA

Based on the introduction of HS and FA in the previous section, the combination of the two approaches is described and HS/FA is proposed, which updates the poor solutions to accelerate its convergence speed.

HS and FA are adept at exploring the search space and exploiting solution, respectively. Therefore, in the present work, a hybrid by inducing HS into FA method named HS/FA is utilized to deal with optimization problem, which can be considered as mutation operator. By this strategy, the mutation of the HS and FA can explore the new search space and exploit the population, respectively. Therefore, it can overcome the lack of the exploration of the FA. 

To combat the random walks used in FA, in the present work, the addition of mutation operator is introduced into the FA, including two detailed improvements. 

The first one is the introduction of top fireflies scheme into FA to reduce running time that is analogous to the elitism scheme frequently used in other population-based optimization algorithms. In FA, due to dual loop, time complexity is O(NP^2^), whose performance significantly deteriorates with the increases in population size. This improvement is carried out by reduction of outer loop in FA. In HS/FA, we select the special firefly with optimal or near-optimal fitness (i.e., the brightest fireflies) to form top fireflies, and all the fireflies only move towards top fireflies. Through top fireflies scheme, the time complexity of HS/FA decreases from O(NP^2^) to O(KEEP∗NP), where KEEP is the number of top fireflies. In general, KEEP is far smaller than NP, so the time used by HS/FA is much less than FA. Apparently, if KEEP = NP, the algorithm HS/FA is declined to the standard FA. If KEEP is too small, only few best fireflies are selected to form top fireflies and it converges too fast, moreover, may be premature for lack of diversity. If KEEP is extremely big (near NP), almost all the fireflies are used to form top fireflies, so all fireflies are explored well, leading to potentially optimal solutions, while the algorithm performs badly and converges too slowly. Therefore, we use KEEP = 2 in our study. 

The second is the addition of HS serving as mutation operator striving to improve the population diversity to avoid the premature convergence. In standard FA, if firefly *i* is brighter than firefly *j*, firefly *j* will move towards firefly *i*, and then evaluate newly-generated fireflies and update light intensity. If not, firefly *j* does nothing. However, in HS/FA, if firefly *i* is not brighter than firefly *j*, firefly *j* is updated by mutation operation to improve the light intensity for firefly *j*. More concretely, for the global search part, with respect to HS/FA, we tune every element *x*_*kj*_ (*k* = 1, 2, …, *D*) in *x*_*j*_ (the position of firefly *j*) using HS. When *ξ*_1_ is not less than HMCR, that is, *ξ*_1_ ≥ HMCR, the element *x*_*kj*_ is updated randomly; whereas when *ξ*_1_ < HMCR, we update the element *x*_*kj*_ in accordance with *x*_*r*1_. Under this circumstance, pitch adjustment operation in HS is applied to update the element *x*_*kj*_ if *ξ*_2_ < PAR to increase population diversity, as shown in (2), where *ξ*_1_ and *ξ*_2_ are two uniformly distributed random numbers in [0,1], **r**_1_ is the integer number in [1, NP], and NP is population size.

In sum, the detailed presentation of HS/FA can be given in Algorithm 3.

## 5. The Results

The HS/FA method is tested on optimization problems through several simulations conducted in test problems. To make a fair comparison between different methods, all the experiments were conducted on the same conditions described in [1].

In this section, HS/FA is compared on optimization problems with other nine methods, which are ACO [11], BBO [40], DE [28–30], ES [9, 10], FA [41, 42], GA [8], HS [17–19], PSO [32, 52], and SGA [36]. Here, for HS, FA, and HS/FA, the parameters are set as follows: absorption coefficient *γ* = 1.0, the HMCR = 0.9, and the PAR = 0.1. For parameters used in other methods, they can be referred to as in [48, 53]. Thirty-six functions are utilized to verify our HS/FA method, which can be shown in Table 1. More knowledge of all the benchmarks can be found in [54].

Because all the intelligent algorithms always have some randomness, in order to get representative statistical features, we did 500 implementations of each method on each problem. Tables 2 and 3 illustrate the average and best results found by each algorithm, respectively. Note that we have used two different scales to normalize the values in the tables, and its detailed process can be found in [54]. The dimension of each function is set to 30.

From Table 2, on average, HS/FA is well capable of finding function minimum on twenty-eight of the thirty-six functions. FA performs the second best on ten of the thirty-six functions.  Table 3 shows that HS/FA and FA perform the same and best on twenty-two of the thirty-six and seventeen functions, respectively. ACO, DE, and GA perform the best on eight benchmarks. From the above tables, we can see that, for low-dimensional functions, both FA and HS/FA perform well, and their performance has little difference between each other.

**Figure 1:**
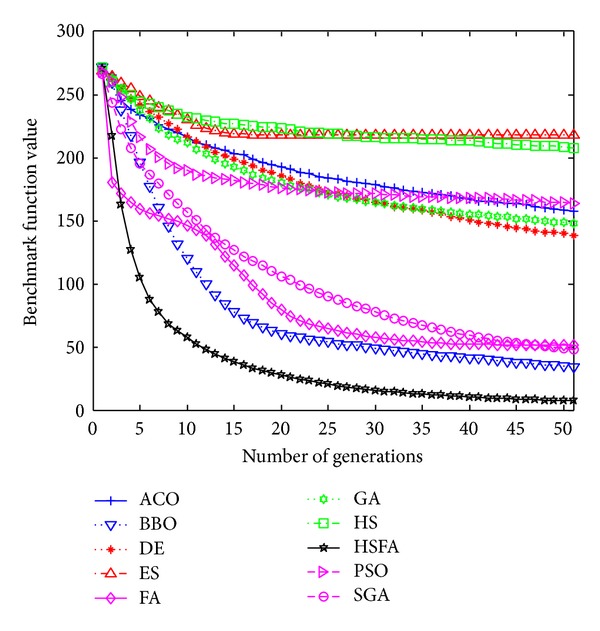
Performance comparison for the F26 Rastrigin function.

**Figure 2:**
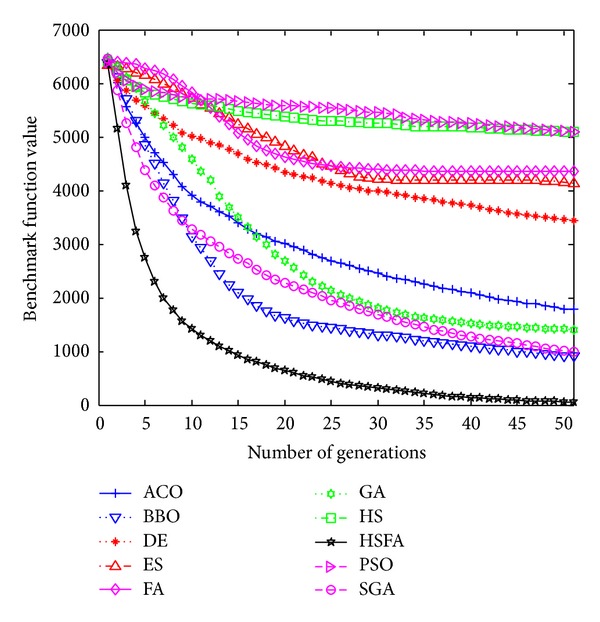
Performance comparison for the F28 Schwefel 2.26 function.

**Figure 3:**
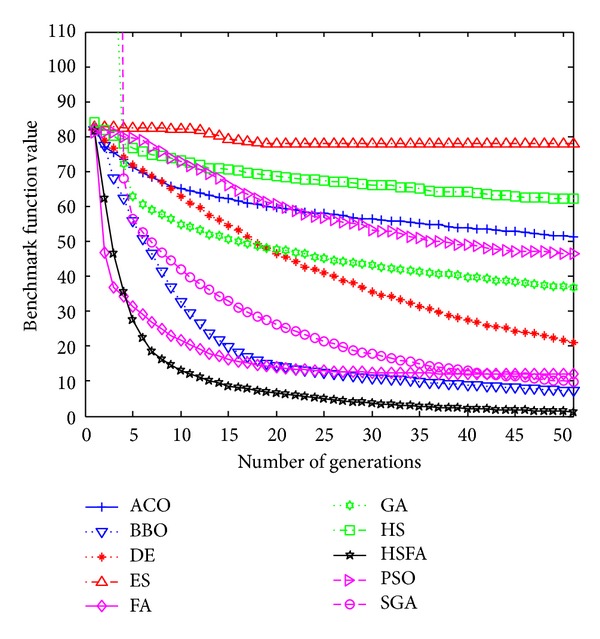
Performance comparison for the F30 Schwefel 2.22 function.

**Figure 4:**
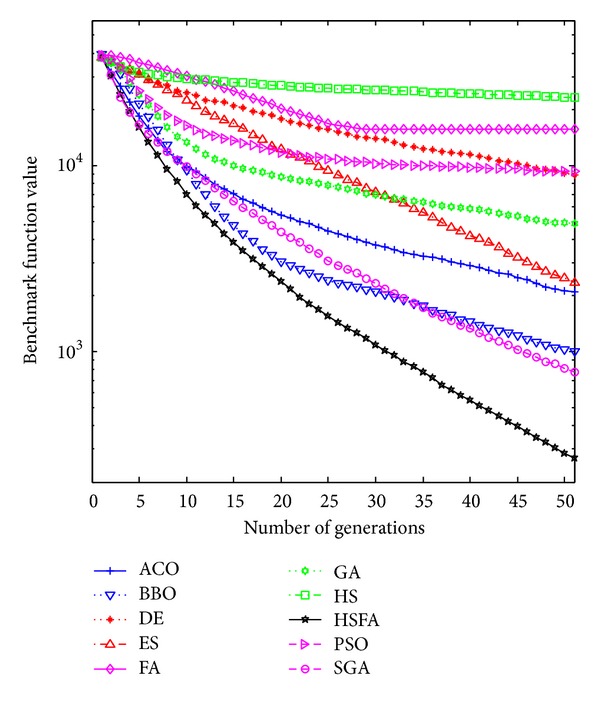
Performance comparison for the F33 step function.

Further, convergence graphs of ten methods for most representative functions are illustrated in Figures 1, 2, 3, and 4 which indicate the optimization process. The values here are the real mean function values from above experiments.

F26 is a complicated multimodal function and it has a single global value 0 and several local optima.  Figure 1 shows that HS/FA converges to global value 0 with the fastest speed. Here FA converges a little faster initially, but it is likely to be trapped into subminima as the function value decreases slightly.

F28 is also a multimodal problem and it has only a global value 0. For this problem, HS/FA is superior to the other nine methods and finds the optimal value earliest. 

For this function, the figure illustrates that HS/FA significantly outperforms all others in the optimization process. At last, HS/FA converges to the best solution superiorly to others. BBO is only inferior to HS/FA and performs the second best for this case.

HS/FA significantly outperforms all others in the optimization process. Furthermore, Figure 4 indicates that, at the early stage of the optimization process, FA converges faster than HS/FA, while HS/FA is well capable of improving its solution steadily in the long run. Here FA shows faster converges initially (within 20 iterations), however it seems to be trapped into subminima as the function value decreases slightly (after 20 iterations), and it is outperformed by HS/FA after 30 iterations. 

From Figures 1–4, our HS/FA's performance is far better than the others. In general, BBO and FA, especially FA, are only inferior to the HS/FA. Note that, in [40], BBO is compared with seven EAs and an engineering problem. The experiments proved the excellent performance of BBO. It is also indirectly proven that our HS/FA is a more effective optimization method than others.

## 6. Conclusions

In the present work, a hybrid HS/FA was proposed for optimization problems. FA is enhanced by the combination of the basic HS method. In HS/FA, top fireflies scheme is introduced to reduce running time; the other is used to mutate between fireflies when updating fireflies. The new harmony vector takes the place of the new firefly only if it is better than before, which generally outperforms HS and FA. The HS/FA strive to exploit merits of the FA and HS so as to escape all fireflies being trapped into local optima. Benchmark evaluation on the test problems is used to investigate the HS/FA and the other nine approaches. The results demonstrated that HS/FA is able to make use of the useful knowledge more efficiently to find much better values compared with the other optimization algorithms.

## Figures and Tables

**Algorithm 1:**
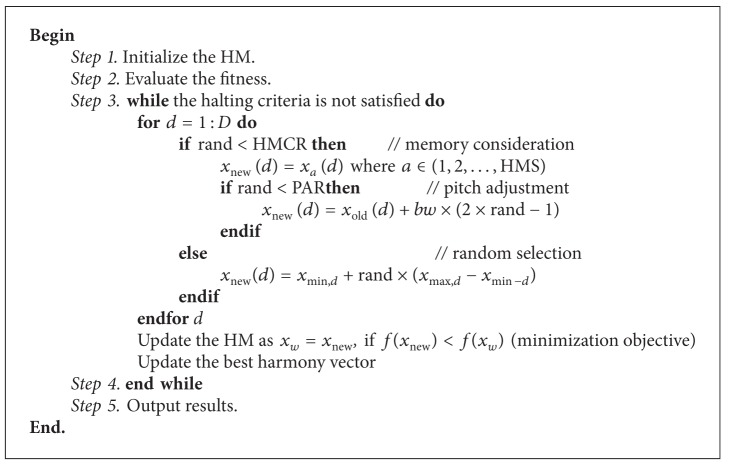
HS method.

**Algorithm 2:**
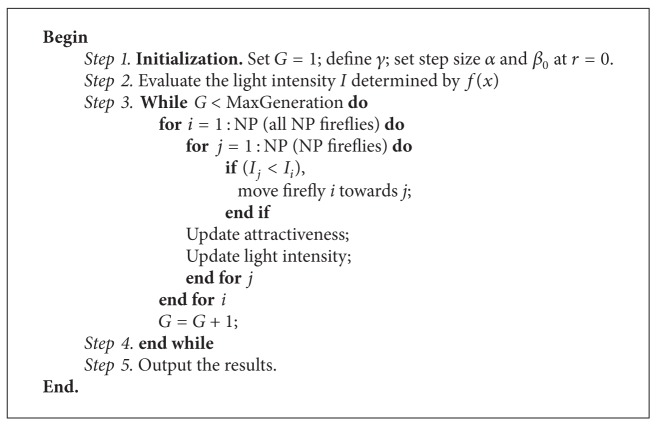
Firefly algorithm. FA method.

**Algorithm 3:**
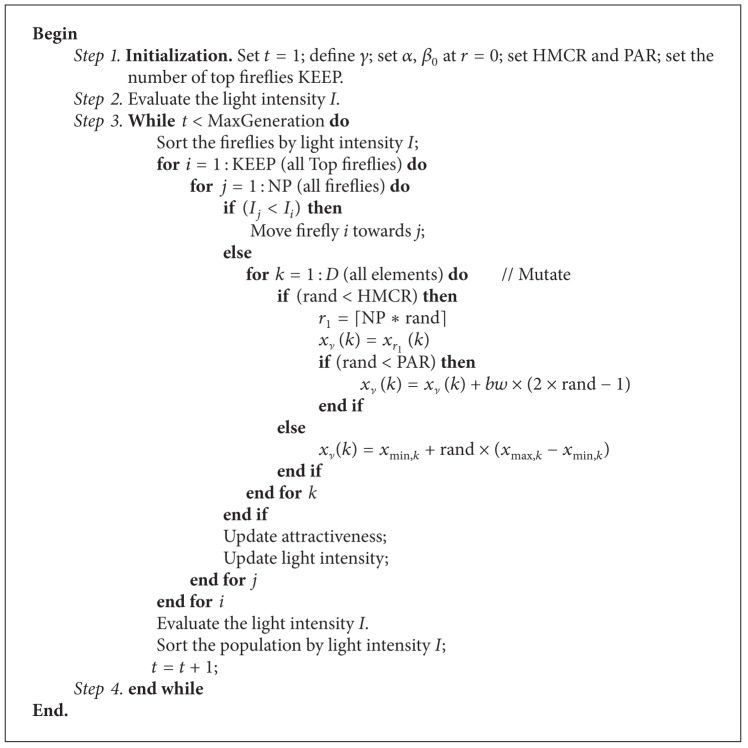
HS/FA method.

**Table 1:** Benchmark functions.

No.	Name	No.	Name
F01	Beale	F19	Holzman 2 function
F02	Bohachevsky #1	F20	Levy
F03	Bohachevsky #2	F21	Pathological function
F04	Bohachevsky #3	F22	Penalty #1
F05	Booth	F23	Penalty #2
F06	Branin	F24	Powel
F07	Easom	F25	Quartic with noise
F08	Foxholes	F26	Rastrigin
F09	Freudenstein-Roth	F27	Rosenbrock
F10	Goldstein-Price	F28	Schwefel 2.26
F11	Hump	F29	Schwefel 1.2
F12	Matyas	F30	Schwefel 2.22
F13	Ackley	F31	Schwefel 2.21
F14	Alpine	F32	Sphere
F15	Brown	F33	Step
F16	Dixon and Price	F34	Sum function
F17	Fletcher-Powell	F35	Zakharov
F18	Griewank	F36	Wavy1

**Table 2:** Mean normalized optimization results.

	ACO	BBO	DE	ES	FA	GA	HS	HSFA	PSO	SGA
F01	1.01	1.01	**1.00**	1.02	1.08	1.04	1.07	**1.00**	1.01	1.25
F02	1.43	2.71	**1.00**	2.55	**1.00**	1.39	16.66	**1.00**	3.13	1.22
F03	1.25	1.84	**1.00**	2.28	**1.00**	1.17	11.77	1.01	3.50	1.26
F04	3.9*E*4	1.7*E*5	49.66	2.8*E*5	**1.00**	4.0*E*4	2.0*E*6	1.5*E*3	3.7*E*5	2.5*E*5
F05	1.01	1.02	**1.00**	1.11	**1.00**	1.01	1.15	**1.00**	1.05	1.19
F06	1.03	1.02	**1.00**	1.09	**1.00**	1.02	1.03	**1.00**	1.03	3.01
F07	2.40	2.48	2.27	2.35	2.23	1.83	1.88	**1.00**	1.71	2.99
F08	1.72	1.72	1.72	1.72	1.72	1.72	1.72	1.72	1.72	**1.00**
F09	1.03	1.01	**1.00**	2.05	17.29	**1.00**	6.55	**1.00**	1.04	1.24
F10	2.40	2.40	2.40	3.06	2.40	2.40	3.09	2.40	2.70	**1.00**
F11	**1.00**	**1.00**	**1.00**	1.03	**1.00**	**1.00**	1.03	**1.00**	1.02	1.25
F12	**1.00**	**1.00**	**1.00**	1.01	**1.00**	**1.00**	1.02	**1.00**	**1.00**	1.14
F13	4.32	2.56	3.68	5.56	1.39	4.98	5.70	**1.00**	4.83	2.63
F14	36.17	7.98	43.16	73.74	11.68	33.13	70.32	**1.00**	53.62	8.48
F15	570.98	14.02	27.90	1.1*E*3	141.86	99.02	652.65	**1.00**	485.92	12.10
F16	1.6*E*3	75.20	317.08	1.2*E*4	7.35	942.08	1.1*E*4	**1.00**	1.6*E*3	26.84
F17	21.98	2.35	7.67	21.63	5.30	7.33	19.01	**1.00**	15.46	2.26
F18	8.49	5.40	14.18	66.70	2.31	28.49	139.02	**1.00**	52.77	5.69
F19	2.8*E*3	167.15	544.18	1.9*E*4	25.71	1.3*E*3	1.9*E*4	**1.00**	2.7*E*3	39.88
F20	93.79	13.59	68.16	276.60	20.05	92.42	282.65	**1.00**	173.17	9.33
F21	3.20	2.49	1.74	**1.00**	3.69	2.65	3.88	1.78	2.55	2.42
F22	1.2*E*8	9.7*E*3	2.8*E*5	5.1*E*7	6.64	5.8*E*5	7.8*E*7	1.00	7.9*E*6	9.81
F23	2.2*E*7	2.9*E*4	3.1*E*5	1.4*E*7	7.36	6.7*E*5	2.2*E*7	1.00	3.5*E*6	5.0*E*3
F24	112.59	8.00	48.08	188.98	1.04	25.76	133.99	**1.00**	52.91	2.92
F25	1.2*E*3	103.34	637.38	1.8*E*4	17.91	1.4*E*3	1.8*E*4	**1.00**	4.1*E*3	62.77
F26	24.37	4.58	21.06	32.51	7.75	20.84	29.89	**1.00**	23.03	7.29
F27	37.23	2.38	5.34	49.70	**1.00**	10.38	34.12	1.04	12.06	2.00
F28	37.76	18.45	73.51	92.92	93.82	31.93	109.20	**1.00**	112.28	21.21
F29	4.79	2.52	6.72	7.41	1.00	5.40	7.13	1.93	4.81	4.31
F30	42.08	6.33	16.76	63.65	9.36	30.16	53.57	**1.00**	35.27	8.32
F31	2.98	3.13	3.87	4.56	**1.00**	3.93	4.78	1.15	3.97	2.79
F32	205.80	13.56	37.41	382.80	1.87	131.27	361.49	**1.00**	151.62	14.65
F33	40.44	20.26	53.05	312.01	5.14	111.20	471.25	**1.00**	194.10	15.72
F34	274.21	27.02	46.57	546.26	6.17	138.85	550.25	**1.00**	188.96	25.75
F35	1.2*E*5	1.46	3.32	3.57	1.18	3.12	3.34	**1.00**	3.12	2.64
F36	9.82	5.26	14.12	29.95	10.67	16.90	35.57	**1.00**	23.95	5.37

The bold data are the best function value among different methods for the specified function.

**Table 3:** Best normalized optimization results.

	ACO	BBO	DE	ES	FA	GA	HS	HSFA	PSO	SGA
F01	**1.00**	**1.00**	**1.00**	**1.00**	**1.00**	**1.00**	**1.00**	**1.00**	**1.00**	**1.00**
F02	**1.00**	1.71	**1.00**	1.53	**1.00**	**1.00**	1.49	**1.00**	1.72	**1.00**
F03	**1.00**	1.28	**1.00**	1.12	**1.00**	**1.00**	1.28	**1.00**	1.34	1.13
F04	2.0*E*14	2.0*E*14	3.2*E*10	2.3*E*15	2.5*E*8	**1.00**	1.1*E*15	3.8*E*11	1.0*E*15	4.5*E*15
F05	**1.00**	**1.00**	**1.00**	1.02	**1.00**	**1.00**	**1.00**	**1.00**	**1.00**	**1.00**
F06	1.01	1.01	**1.00**	1.01	**1.00**	1.01	**1.00**	**1.00**	**1.00**	2.51
F07	2.5*E*6	3.3*E*6	7.2*E*5	1.6*E*6	**1.00**	1.7*E*4	2.1*E*5	2.88	3.7*E*5	3.3*E*6
F08	1.99	1.99	1.99	1.99	1.99	1.99	1.99	1.99	1.99	**1.00**
F09	**1.00**	**1.00**	**1.00**	1.01	**1.00**	**1.00**	**1.00**	**1.00**	**1.00**	**1.00**
F10	2.65	2.65	2.65	2.74	2.65	2.65	2.65	2.65	2.65	**1.00**
F11	**1.00**	**1.00**	**1.00**	**1.00**	**1.00**	**1.00**	**1.00**	**1.00**	**1.00**	1.03
F12	**1.00**	**1.00**	**1.00**	**1.00**	**1.00**	**1.00**	**1.00**	**1.00**	**1.00**	**1.00**
F13	8.34	3.93	7.05	11.02	**1.00**	8.71	11.56	1.54	9.51	3.98
F14	63.46	11.42	88.82	159.53	12.22	40.65	144.04	**1.00**	102.48	10.66
F15	218.30	9.54	47.47	591.91	33.50	128.64	792.73	**1.00**	358.64	9.44
F16	4.7*E*3	109.24	1.3*E*3	4.0*E*4	**1.00**	315.53	6.1*E*4	2.31	5.9*E*3	43.61
F17	42.58	3.24	11.83	41.06	1.20	9.50	43.49	**1.00**	29.07	3.02
F18	7.99	3.62	13.85	63.26	**1.00**	14.42	160.01	1.08	37.87	2.32
F19	3.1*E*3	135.16	893.82	3.9*E*4	1.56	566.65	5.4*E*4	**1.00**	6.1*E*3	3.67
F20	251.29	32.94	142.30	720.64	7.96	131.48	863.43	**1.00**	483.70	23.66
F21	3.96	2.89	1.65	**1.00**	4.60	2.91	4.87	1.90	2.72	2.37
F22	31.83	55.55	1.5*E*5	1.3*E*8	8.26	89.30	3.0*E*8	**1.00**	5.8*E*6	15.15
F23	**1.00**	4.4*E*3	1.5*E*6	8.8*E*7	27.10	3.3*E*4	1.6*E*8	4.89	2.8*E*7	29.22
F24	2.2*E*3	88.70	1.1*E*3	4.7*E*3	1.60	215.01	3.4*E*3	**1.00**	940.82	34.50
F25	3.0*E*3	380.11	2.9*E*3	1.0*E*5	**1.00**	3.1*E*3	1.3*E*5	2.01	2.4*E*4	54.67
F26	39.66	5.65	30.04	58.39	6.03	27.36	42.32	**1.00**	38.57	8.91
F27	54.77	2.13	12.53	87.36	1.27	10.85	58.14	**1.00**	21.11	2.77
F28	164.45	67.82	335.75	447.01	430.29	85.82	596.00	**1.00**	551.44	68.85
F29	8.78	4.00	16.50	15.31	**1.00**	10.85	18.42	3.27	6.75	7.29
F30	63.53	10.38	27.71	105.79	7.15	47.04	88.91	**1.00**	58.02	12.83
F31	3.80	5.63	7.23	9.39	**1.00**	7.31	10.20	1.93	7.56	4.53
F32	740.24	30.59	184.72	1.8*E*3	**1.00**	322.80	1.8*E*3	2.87	725.62	31.00
F33	149.29	66.43	224.57	1.4*E*3	3.86	255.71	2.4*E*3	**1.00**	1.0*E*3	42.71
F34	491.51	35.62	100.26	1.1*E*3	1.64	113.66	1.1*E*3	**1.00**	400.61	27.55
F35	3.44	2.50	5.89	6.67	**1.00**	4.28	5.48	1.46	3.83	3.74
F36	11.05	6.01	18.56	40.10	9.07	18.47	43.18	**1.00**	31.18	4.64

The bold data are the best function value among different methods for the specified function.

## References

[1] Wang G, Guo L (2013). A novel hybrid bat algorithm with harmony search for global numerical optimization. *Journal of Applied Mathematics*.

[2] Li X, Yin M (2013). An opposition-based differential evolution algorithm for permutation flow shop scheduling based on diversity measure. *Advances in Engineering Software*.

[3] Zou D, Gao L, Li S, Wu J (2011). An effective global harmony search algorithm for reliability problems. *Expert Systems with Applications*.

[4] Zou D, Gao L, Wu J, Li S, Li Y (2010). A novel global harmony search algorithm for reliability problems. *Computers and Industrial Engineering*.

[5] Yang X-S, Cui Z, Xiao R, Gandomi AH, Karamanoglu M (2013). *Swarm Intelligence and Bio-Inspired Computation*.

[6] Gandomi AH, Yang XS, Talatahari S, Alavi AH (2013). *Metaheuristic Applications in Structures and Infrastructures*.

[7] Yang XS, Gandomi AH, Talatahari S, Alavi AH (2013). *Metaheuristics in Water, Geotechnical and Transport Engineering*.

[8] Goldberg DE (1989). *Genetic Algorithms in Search, Optimization and Machine Learning*.

[9] Back T (1996). *Evolutionary Algorithms in Theory and Practice*.

[10] Beyer H (2001). *The Theory of Evolution Strategies*.

[11] Dorigo M, Stutzle T (2004). *Ant Colony Optimization*.

[12] Shumeet B (1994). Population-based incremental learning: a method for integrating genetic search based function optimization and competitive learning. *Carnegie Mellon University*.

[13] Erol OK, Eksin I (2006). A new optimization method: big bang-big crunch. *Advances in Engineering Software*.

[14] Kaveh A, Talatahari S (2009). Size optimization of space trusses using big bang-big crunch algorithm. *Computers and Structures*.

[15] Kaveh A, Talatahari S (2010). Optimal design of schwedler and ribbed domes via hybrid big bang-big crunch algorithm. *Journal of Constructional Steel Research*.

[16] Kaveh A, Talatahari S (2010). A discrete big bang-big crunch algorithm for optimal design of skeletal structures. *Asian Journal of Civil Engineering*.

[17] Geem ZW, Kim JH, Loganathan GV (2001). A new heuristic optimization algorithm: harmony search. *Simulation*.

[18] Yadav P, Kumar R, Panda SK, Chang CS (2012). An intelligent tuned harmony search algorithm for optimisation. *Information Sciences*.

[19] Gholizadeh S, Barzegar A (2013). Shape optimization of structures for frequency constraints by sequential harmony search algorithm. *Engineering Optimization*.

[20] Kaveh A, Talatahari S (2010). A novel heuristic optimization method: charged system search. *Acta Mechanica*.

[21] Xie L, Zeng J, Formato RA (2012). Selection strategies for gravitational constant G in artificial physics optimisation based on analysis of convergence properties. *International Journal of Bio-Inspired Computation*.

[22] Gandomi AH, Yang X-S, Alavi AH, Talatahari S (2013). Bat algorithm for constrained optimization tasks. *Neural Computing & Applications*.

[23] Yang XS, Gandomi AH (2012). Bat algorithm: a novel approach for global engineering optimization. *Engineering Computations*.

[24] Li X, Zhang J, Yin M (2013). Animal migration optimization: an optimization algorithm inspired by animal migration behavior. *Neural Computing and Applications*.

[25] Gandomi AH, Alavi AH (2012). Krill herd: a new bio-inspired optimization algorithm. *Communications in Nonlinear Science and Numerical Simulation*.

[26] Wang G-G, Gandomi AH, Alavi AH (2013). Stud krill herd algorithm. *Neurocomputing*.

[27] Wang G-G, Gandomi AH, Alavi AH (2013). An effective krill herd algorithm with migration operator in biogeography-based optimization. *Applied Mathematical Modelling*.

[28] Storn R, Price K (1995). Differential evolution-a simple and efficient adaptive scheme for global optimization over continuous spaces.

[29] Storn R, Price K (1997). Differential evolution-a simple and efficient heuristic for global optimization over continuous spaces. *Journal of Global Optimization*.

[30] Li X, Yin M (2012). Application of differential evolution algorithm on self-potential data. *PLoS One*.

[31] Wang GG, Gandomi AH, Alavi AH, Hao GS (2013). Hybrid krill herd algorithm with differential evolution for global numerical optimization. *Neural Computing & Applications*.

[32] Kennedy J, Eberhart R Particle swarm optimization.

[33] Kuo RJ, Syu YJ, Chen Z-Y, Tien FC (2012). Integration of particle swarm optimization and genetic algorithm for dynamic clustering. *Information Sciences*.

[34] Talatahari S, Kheirollahi M, Farahmandpour C, Gandomi AH (2013). A multi-stage particle swarm for optimum design of truss structures. *Neural Computing & Applications*.

[35] Huang KY (2011). A hybrid particle swarm optimization approach for clustering and classification of datasets. *Knowledge-Based Systems*.

[36] Khatib W, Fleming P (1998). The stud GA: a mini revolution?. *Parallel Problem Solving from Nature*.

[37] Yang XS, Deb S Cuckoo search via Lévy flights.

[38] Gandomi AH, Talatahari S, Yang XS, Deb S (2013). Design optimization of truss structures using cuckoo search algorithm. *The Structural Design of Tall and Special Buildings*.

[39] Cai X, Fan S, Tan Y (2012). Light responsive curve selection for photosynthesis operator of APOA. *International Journal of Bio-Inspired Computation*.

[40] Simon D (2008). Biogeography-based optimization. *IEEE Transactions on Evolutionary Computation*.

[41] Gandomi AH, Yang X-S, Alavi AH (2011). Mixed variable structural optimization using firefly algorithm. *Computers & Structures*.

[42] Yang XS (2008). *Nature-Inspired Metaheuristic Algorithms*.

[43] Yang XS Firefly algorithms for multimodal optimization.

[44] Yang XS (2010). Firefly algorithm, stochastic test functions and design optimisation. *International Journal of Bio-Inspired Computation*.

[45] Yang X-S, Hosseini SSS, Gandomi AH (2012). Firefly algorithm for solving non-convex economic dispatch problems with valve loading effect. *Applied Soft Computing Journal*.

[46] Parpinelli R, Lopes H (2011). New inspirations in swarm intelligence: a survey. *International Journal of Bio-Inspired Computation*.

[47] Zou D, Gao L, Wu J, Li S (2010). Novel global harmony search algorithm for unconstrained problems. *Neurocomputing*.

[48] Wang G, Guo L, Wang H, Duan H, Liu L, Li J (2012). Incorporating mutation scheme into krill herd algorithm for global numerical optimization. *Neural Computing and Applications*.

[49] Zhao SZ, Suganthan PN, Pan Q-K, Fatih Tasgetiren M (2011). Dynamic multi-swarm particle swarm optimizer with harmony search. *Expert Systems with Applications*.

[50] Wang G, Guo L, Duan H, Liu L, Wang H (2012). A modified firefly algorithm for UCAV path planning. *International Journal of Hybrid Information Technology*.

[51] Gandomi AH, Yang XS, Talatahari S, Alavi AH (2013). Firefly algorithm with chaos. *Communications in Nonlinear Science and Numerical Simulation*.

[52] Zhang Y, Huang D, Ji M, Xie F (2011). Image segmentation using PSO and PCM with Mahalanobis distance. *Expert Systems with Applications*.

[53] Wang GG, Guo L, Gandomi AH, Alavi AH, Duan H (2013). Simulated annealing-based krill herd algorithm for global optimization. *Abstract and Applied Analysis*.

[54] Yao X, Liu Y, Lin G (1999). Evolutionary programming made faster. *IEEE Transactions on Evolutionary Computation*.

